# Direct prion neuroinvasion following inhalation into the nasal cavity

**DOI:** 10.1128/msphere.00863-24

**Published:** 2024-11-29

**Authors:** Anthony E. Kincaid, Melissa D. Clouse, Shawn M. Magrum, Jason C. Bartz

**Affiliations:** 1 Department of Pharmacy Sciences, School of Pharmacy and Health Professions, Creighton University, Omaha, Nebraska, USA; 2 Department of Biomedical Sciences, School of Medicine, Creighton University, Omaha, Nebraska, USA; 3 Department of Medical Microbiology and Immunology, School of Medicine, Creighton University, Omaha, Nebraska, USA; University of Michigan, Ann Arbor, Michigan, USA

**Keywords:** prion disease, neuroinvasion, prion diagnostics, nasal cavity, trigeminal ganglia, trigeminal nerve

## Abstract

Inhalation of prions into the nasal cavity is an efficient route of infection. Following inhalation of infectious prions, animals develop disease with a similar incubation period compared with per os exposure, but with greater efficiency. To identify the reason for this increased efficiency, we identified neural structures that uniquely innervate the nasal cavity and neural structures known to mediate neuroinvasion following oral infection and used immunohistochemistry to determine the temporal and spatial accumulation of prions from hamster tissue sections containing cell bodies and axons at 2-week intervals following prion exposure. Prions were identified in the trigeminal ganglion, the spinal trigeminal tract in the brainstem, the intermediolateral cell column of the thoracic spinal cord, and the dorsal motor nucleus of the vagus/solitary nucleus complex months prior to detection of prions in the olfactory bulb or superior cervical ganglion. These results indicate that the trigeminal nerve, but not the olfactory nerve or sympathetic nerves, are involved in neuroinvasion following inhalation of prions into the nasal cavity. The detection of prions in the intermediolateral cell column of the thoracic spinal cord and dorsal motor nucleus of the vagus nerve 14 weeks following inhalation is consistent with inoculum crossing the alimentary wall and infecting the enteric nervous system via this route of infection. Neuroinvasion via the trigeminal nerve, in combination with entry into the central nervous system via autonomic innervation of the enteric nervous system, may contribute to increased efficiency of nasal cavity exposure to prions compared with per os exposure in hamsters.

**IMPORTANCE:**

Inhalation of prions into the nasal cavity is thought to be a route of infection in naturally acquired prion diseases. Experimental studies indicate that inhalation of prions is up to two orders of magnitude more efficient compared with ingestion. The mechanisms underlying this observation are poorly understood. We found a previously unreported direct route of neuroinvasion from the nasal cavity to the nervous system. Importantly, the peripheral ganglia involved may be a useful tissue to sample for prion diagnostics. Overall, identification of a new route of neuroinvasion following prion infection may provide an anatomical basis to explain the increased efficiency of infection following prion inhalation.

## INTRODUCTION

Hamsters, mice, sheep, and deer develop prion disease following inhalation of infectious prions (PrP^Sc^) via the nose [for review see Reference (1)]. This route of infection is 10–100 times more efficient than the per os route (2, 3), suggesting that there may be a unique neuroinvasion entry point for PrP^Sc^ in this route of exposure. A comprehensive understanding of the nerves mediating prion neuroinvasion following inhalation into the nasal cavity (NC) is lacking. An obvious potential route of neuroinvasion following inhalation into the nasal cavity is the olfactory nerve (CN I). To date, there is no evidence that prions enter the central nervous system (CNS) via CN I structures following inhalation in experimental animals (2, 4, 5) or in animals that naturally acquire prion disease (6, 7). Other candidate NC nerves that have not been examined but could potentially transmit PrP^Sc^ into the CNS include branches of the trigeminal nerve (CN V) that supply somatic sensation of the mucosa and/or sympathetic nerves, which innervate blood vessels in the submucosa and lymphoid tissues in the head (8–10). This study was designed to determine the spatial and temporal spread of prions in nerves and ganglia associated with the NC following inhalation and identify which structures are involved in the centripetal spread of prions. To this end, hamsters were inoculated with HY TME-infected or mock-infected brain homogenate (bh), and neural tissues associated with the NC and per os route of infection were collected at 2-week intervals up to 22 weeks post-infection following inhalation of prions and subsequently processed for the presence of PrP^Sc^ using immunohistochemistry (IHC). Unrelated data from these animals were used in a previous publication (2).

## RESULTS

### Specificity of PrP^Sc^ immunoreactivity

**Fig 1:**
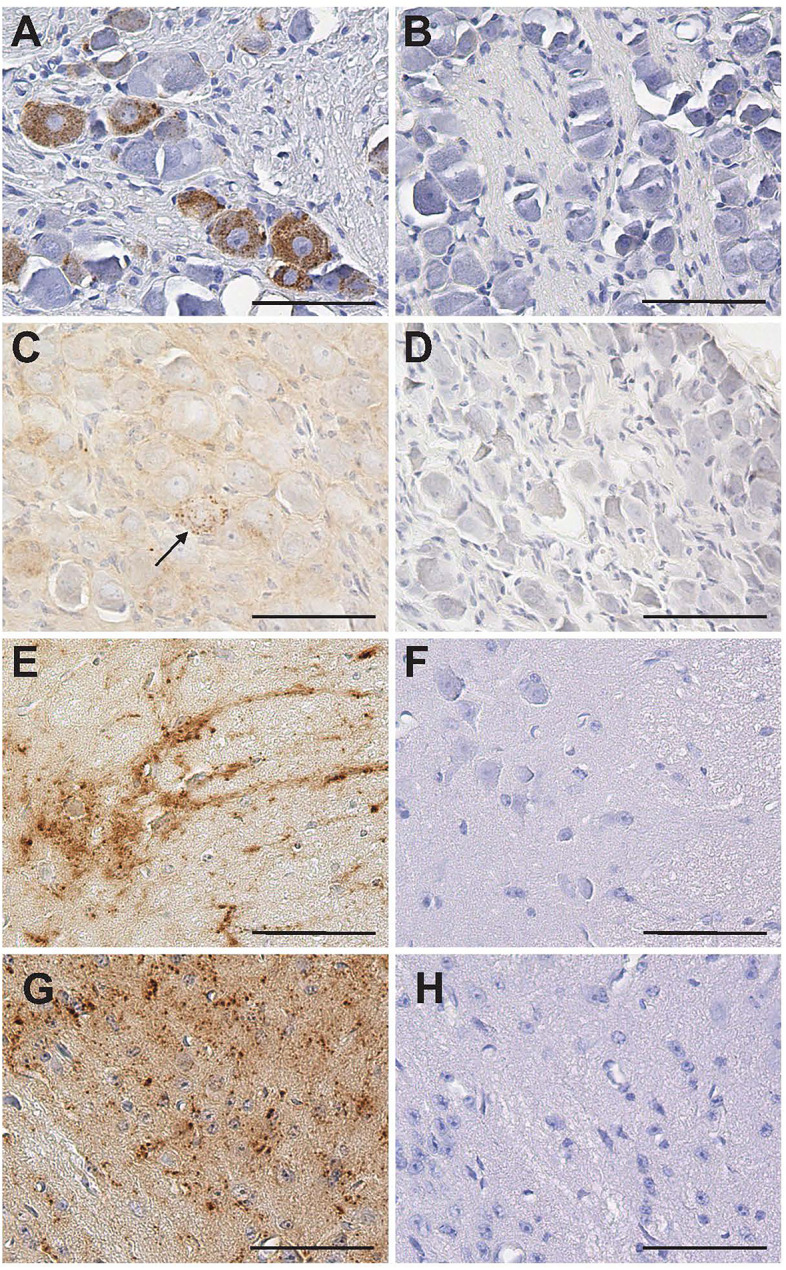
Detection of PrP^Sc^ immunoreactivity (ir) following inhalation of prions into the nasal cavity (NC) at 22 weeks post-inoculation. There was robust PrP^Sc^-ir in a subpopulation of neurons in the trigeminal (**V**) ganglion (panel A) following inhalation of infected brain homogenate (bh) but was not detected following inhalation of mock-infected bh (panel B). There was a small number of PrP^Sc^-ir neurons in the superior cervical ganglion following inhalation of infected bh (indicated by arrow in panel C) and no detectable ir following inhalation of mock-infected bh (panel D). PrP^Sc^-ir was pronounced in neurons in the intermediolateral cell column (ICC) of the thoracic spinal cord following inhalation (panel E); there was no detectable ir in the ICC of the spinal cord (panel F) when adjacent tissue sections were processed using an isotype control. The dorsal motor nucleus of the vagus and solitary nucleus contained PrP^Sc^-ir following inhalation of infected bh; a medium power view shows widespread PrP^Sc^-ir in the SN (panel G); PrP^Sc^-ir was not detected when an isotope control antibody was utilized on an adjacent section. All scale bars = 50 µm.

PrP^Sc^ immunoreactivity (PrP^Sc^-ir) was compared between mock-infected and prion-infected animals for each tissue, at each time point, to establish the presence of PrP^Sc^. Mock-infected and infected tissues for each time point were processed at the same time, utilizing the same reagents. Regularly spaced tissue sections for each structure at each time point were examined with a light microscope by an investigator (AEK) who was blind to the identity of the animal. Examples of PrP^Sc^-ir in tissues were photographed for each structure at each time point. PrP^Sc^-ir was not detected in any of the tissues collected from the animals inoculated with mock-infected bh at any time point (Fig. 1B and D). PrP^Sc^-ir was characterized by discrete, punctate brown deposits in the cytoplasm of cells or associated with nerves/tracts (Fig. 1A, C, E and G). Omission of the primary or secondary antibodies or replacement of the primary antibody with an isotype control resulted in a lack of PrP^Sc^-ir (Fig. 1F and H).

### CN V involvement in neuroinvasion

**Fig 2:**
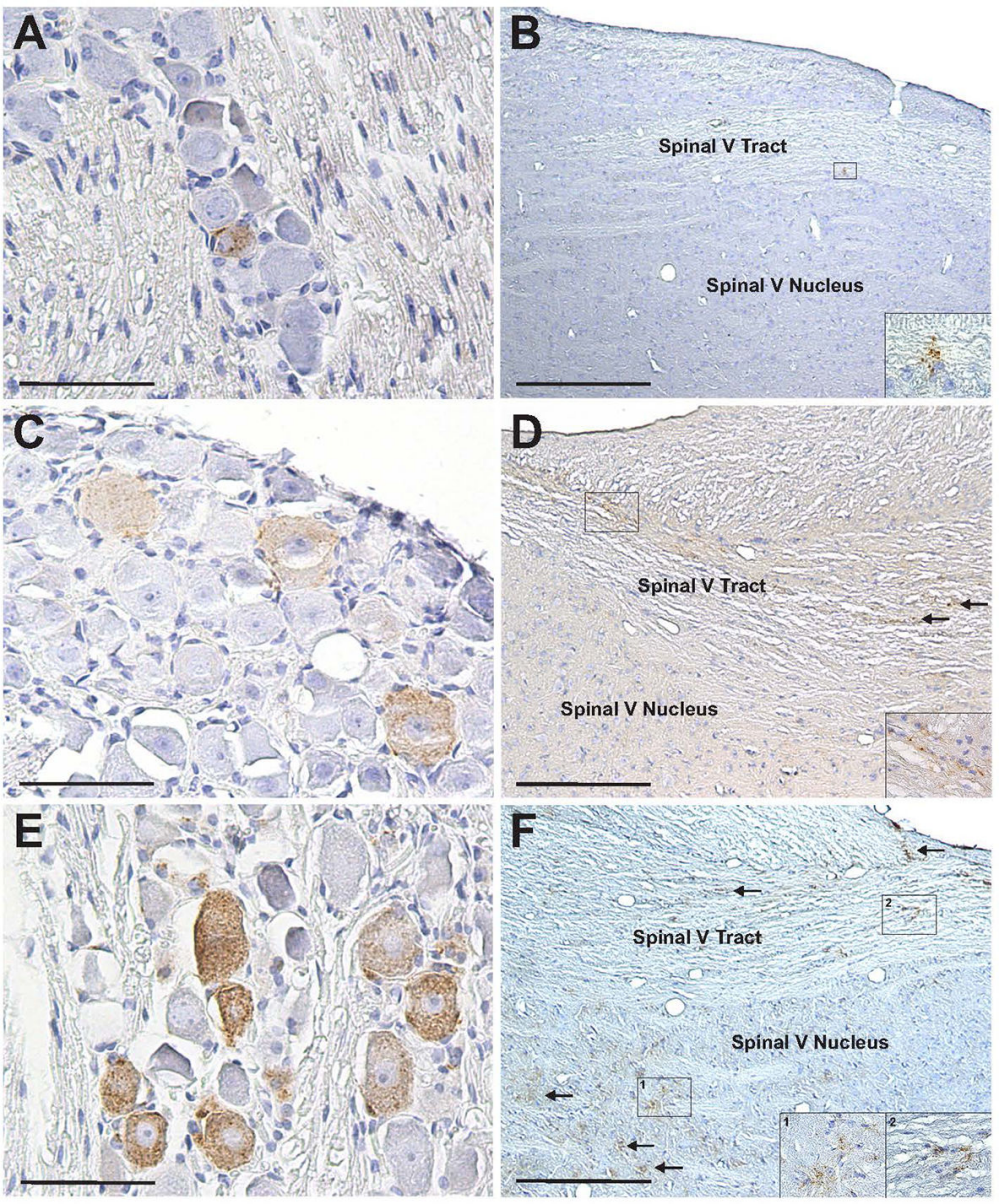
PrP^Sc^-ir in CN V ganglia and V structures in the brainstem increases over time following inhalation of prions. There was an increase in the number of PrP^Sc^-ir CN V ganglion neurons and in the amount of intraneuronal PrP^Sc^-ir from 10 weeks (panel A) to 14 weeks (panel C) to 18 weeks (panel E) following inhalation of infected bh. The same pattern of increased PrP^Sc^-ir was seen in the spinal V tract and spinal V nucleus at 10 (panel B), 14 (panel D), and 18 weeks (panel F) following inhalation of infected bh. The insets in panels B, D, and F are enlarged views of the areas within the boxes showing punctate PrP^Sc^ depositions. The arrows in panels D and F indicate additional areas of PrP^Sc^ deposition following inhalation of infected bh. Scale bars in panels A, C, E = 50 µm; scale bars in panels B, D, F = 200 µm.

PrP^Sc^ was detected in CN V ganglia and spinal V tracts beginning in week 8 post-inoculation and was present in these structures at all subsequent time points (Table 1). PrP^Sc^-ir was detected in spinal V nucleus beginning at week 12 and was also present at all subsequent time points. There was an increase in immunopositive elements in CN V ganglia collected from animals with longer survival times following inoculation (Fig. 2). CN V ganglia contain neuronal cell bodies whose peripheral processes provide somatosensory innervation to the mucosa of the NC, and whose central processes form the spinal V tract and terminate in the spinal V nucleus in the medulla (11). Similar to the CN V ganglia, there was an increase in PrP^Sc^ immunoreactivity in the spinal V tract and spinal V nucleus over time (Fig. 2). The initial detection of PrP^Sc^ in the CN V ganglia and spinal V tract at 8 weeks is 30% of the incubation period for this strain, dose, and route of inoculation (average incubation period of 187 days) (2) and represents the earliest detection of PrP^Sc^ in any nerve or sensory ganglion associated with innervation of the NC.

**TABLE 1:** The presence of PrP^Sc^ in tissues following inhalation of infected brain homogenate^*a*^

	PrP^Sc^ immunoreactivity in anatomical location						
	Olfactory bulb	Trigeminal ganglion	Spinal trigeminal tract	Spinal trigeminal nucleus	Superior cervical ganglion	Spinal cord	Dorsal motor nucleus of vagus/solitary nucleus
Week 6							
6099.1	No	No	No	No	NA	NA	No
6099.2	No	No	No	No	NA	No	No
6099.3	No	No	No	No	NA	No	No
Week 8							
6099.4	No	No	No	No	No	No	No
6099.5	No	Yes	Yes	No	No	No	No
6099.6	No	No	No	No	No	No	No
Week 10							
6100.1	No	Yes	Yes	No	No	No	No
6100.2	No	Yes	Yes	No	No	No	No
6100.3	No	Yes	No	No	No	No	No
Week 12							
6100.4	No	Yes	Yes	Yes	No	No	No
6100.5	No	Yes	Yes	Yes	NA	No	No
6100.6	No	No	Yes	No	NA	No	No
Week 14							
6101.1	No	Yes	Yes	Yes	NA	Yes	Yes
6101.2	No	Yes	Yes	Yes	NA	Yes	No
6101.3	No	Yes	Yes	Yes	NA	Yes	Yes
Week 16							
6101.4	No	Yes	Yes	Yes	No	Yes	Yes
6101.5	No	Yes	Yes	Yes	NA	Yes	Yes
6101.6	No	Yes	Yes	Yes	No	Yes	Yes
Week 18							
6102.1	No	Yes	Yes	Yes	NA	Yes	Yes
6102.2	No	Yes	Yes	Yes	NA	Yes	Yes
6102.3	No	Yes	Yes	Yes	No	Yes	No
Week 20							
6102.4	No	Yes	Yes	Yes	No	Yes	Yes
6102.5	No	Yes	Yes	Yes	NA	Yes	Yes
6102.6	No	Yes	Yes	Yes	NA	Yes	Yes
Week 22							
6103.1	No	Yes	Yes	Yes	Yes	Yes	Yes
6103.2	No	Yes	Yes	Yes	NA	Yes	Yes
6103.3	Yes	Yes	Yes	Yes	Yes	Yes	Yes

^*a*^ NA, not available.

### Olfactory nerve involvement in neuroinvasion

The olfactory bulbs (OBs) are the location of the axon terminals of olfactory sensory neurons located in the olfactory mucosa of the NC. PrP^Sc^ was not detected in OBs until week 22 post-infection following NC exposure (Table 1). This time point is 85% of the incubation period for this dose and route of inoculation (2).

### Sympathetic nerve involvement in neuroinvasion

PrP^Sc^ was not detected in the cells of the superior cervical ganglia (SCG) until week 22 following NC exposure (Table 1; Fig. 1). Only 1–2 SCG neurons per tissue section were PrP^Sc^-positive (Fig. 1C). Similar to the timing of PrP^Sc^ deposition in OBs, the detection of PrP^Sc^ in the SCG was relatively late in the incubation period (85%).

### Spinal cord and dorsal motor nucleus of vagus/solitary nucleus involvement in neuroinvasion

**Fig 3:**
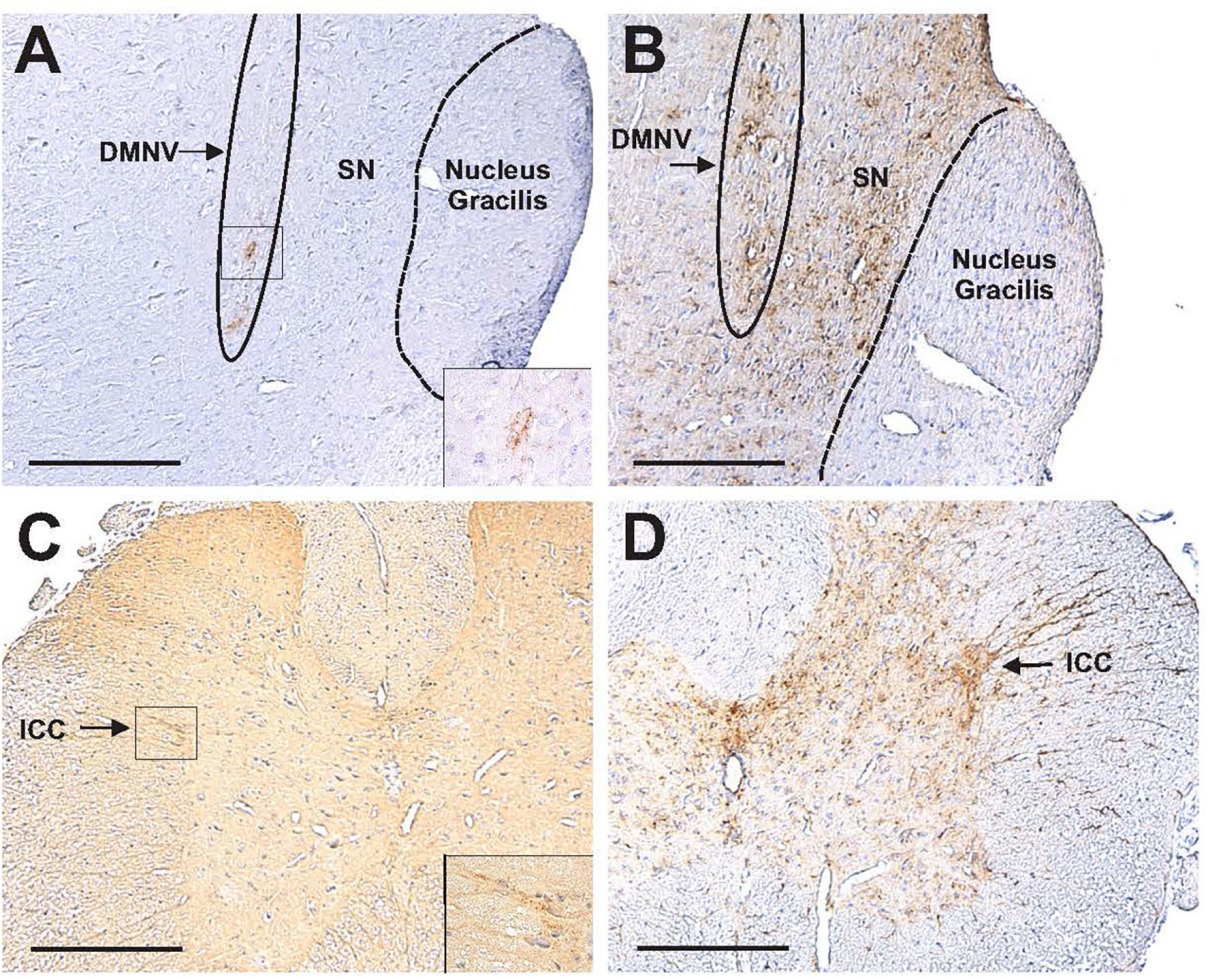
Detection of PrP^Sc^ in the ICC of the thoracic spinal cord and DMNV/SN following inhalation of infected bh into the NC. PrP^Sc^-ir was initially restricted to the DMNV of the medulla (panel A) and to a small number of neurons in the ICC of the thoracic spinal cord (panel C) at 14 weeks following inhalation of infected bh. PrP^Sc^-ir spread noticeably in the DMNV and SN and to adjacent areas (panel B) and spread throughout the gray matter of the thoracic spinal cord (panel D) at 18 weeks post-inhalation. Note that the brain/brainstem sections were cut in the sagittal plane; rostral is toward the top of the figures, and the dorsal surface of the medulla is to the right. The solitary nucleus was identifiable as a relatively narrow tubular structure elongated in the rostral-caudal plane, ventral to the nucleus gracilis (NG), and dorsal to the DMNV. Insets in panels A and C are enlarged views of the areas within the boxes showing the relatively modest amount of PrP^Sc^-ir at 14 weeks post-inhalation. Scale bars = 200 µm.

PrP^Sc^ was initially detected in the intermediolateral cell column (ICC) of the thoracic spinal cord and the dorsal motor nucleus of the vagus nerve (DMNV) and solitary nucleus (SN) beginning at week 14 and was present in these areas at all subsequent time points (Table 1; Fig. 3). The ICC of the thoracic spinal cord is the location of sympathetic preganglionic neurons synaptically linked to postganglionic neurons located in the SCG, which innervate the NC (12). The DMNV/SN are part of the vagal nuclei in the medulla and are the location of parasympathetic preganglionic neurons (DMNV) and sensory neurons (SN) of the vagus nerve that are synaptically linked to neurons that innervate the enteric nervous system (13).

## DISCUSSION

Olfactory and sympathetic nerves do not appear to be involved in the centripetal spread of prions into the hamster CNS following inhalation of infected bh into the NC. The lack of CN I involvement in the neuroinvasion of prions following inhalation, as shown by a lack of PrP^Sc^ in OBs reported here, is consistent with results of previous studies that fail to show PrP^Sc^ deposition in olfactory receptor neurons or OBs following NC exposure until late in the incubation period (2–4). These results also indicate that prions are not likely to be shed in nasal secretions by naturally infected animals until very late in the incubation period just prior to the onset of clinical signs of disease, due to the late spread of PrP^Sc^ to the OBs. The presence of only a small number of PrP^Sc^-ir neurons in the SCG that are not detectable until late in the incubation period indicates that sympathetic fibers that innervate the NC do not contribute to prion neuroinvasion. The PrP^Sc^ that was detected in OBs and SCG at 22 weeks post-inoculation is likely due to the centrifugal spread of prions and not centripetal spread.

The relatively early identification of PrP^Sc^ in CN V ganglia and the spinal V tract, and the progressive increase and spread of PrP^Sc^ in CN V structures over time, indicates that CN V is involved in neuroinvasion following NC exposure. This route of neuroinvasion is not possible in per os exposure as the trigeminal nerve does not innervate the gut. Inoculation of prions via the NC is 10–100 times more efficient than per os inoculation (a lower dose causes disease), and this increase in efficiency may be due to the neuroinvasion route via CN V that is unique to NC exposure and would be expected to occur following either high or low dose extranasal prion exposure. In a previous study, 263K was intranasally inoculated into hamsters, and PrP^Sc^ was detected in CN V nucleus and DMNV/SN in some animals beginning at 100 days post-inoculation (60% of the incubation period) (4). These results are similar to the findings of this study, except for the earlier detection of PrP^Sc^ in the Vth ganglion and Vth tract/nucleus reported here. A potential explanation for this discrepancy in the temporal detection of PrP^Sc^ is the use of different prion strains in the two experiments and/or the use of different sampling strategies. Support for the transport of prions into the CNS following NC exposure comes from studies that have identified transganglionic CN V transport of herpes simplex virus I from tooth pulp into the spinal V nucleus of the medulla and from studies of intranasal injections of insulin that travel via components of CN V to the brain (14, 15).

The presence of PrP^Sc^ in the ICC of the thoracic spinal cord (beginning at week 14) does not appear to be the result of transport via sympathetic fibers that innervate the NC, as PrP^Sc^ was not detectable in the SCG until week 22. The detection of PrP^Sc^ in both the ICC and DMNV shown here is similar to what was reported when 263K was experimentally fed to hamsters (16). Thus, the PrP^Sc^ in the ICC reported here is likely due to retrograde transport of prions via sympathetic nerves that innervate the enteric nervous system, which was a result of inhaled inoculum passing down the nasopharynx to enter the alimentary tract.

There are several important advantages to studies that utilize inoculations of prions in experimental animals versus studies of naturally infected animals. The first is the ability to collect and analyze relevant tissues at regular intervals post-inoculation, which allows for the differentiation of neuroinvasion (centripetal spread) from centrifugal spread (PrP^Sc^ transported from the CNS to the periphery). Another advantage is the potential ability to compare the relative effects of different doses and routes of infection on incubation period, routes of neuroinvasion, and patterns of spread within the CNS. An advantage of using the hamster model of infection is the smaller size of all relevant structures compared with natural host species. This allows for the processing and analysis of a larger proportion of each structure, reducing the chance of missing early indications of disease and increasing the rigor of the studies. Finally, although IHC and light microscopy are not the most sensitive and efficient tools to detect small amounts of PrP^Sc^, their use does preserve anatomical detail that allows for the accurate localization of PrP^Sc^ to specific brain areas and cell types, and thus the ability to pinpoint the location of PrP^Sc^ (17–21). The ability to distinguish specific brain nuclei and tracts was required in this study as the spinal V tract and nucleus extend through much of the rostral-caudal extent of the medulla, as does the DMNV and SN. Tissue homogenization, which is used in some detection techniques, would have obscured this observation.

We have identified CN V as a relatively early and potential route of prion entry into the CNS following NC exposure. PrP^Sc^ was detected in neurons of CN V ganglia, in the central processes of these neurons in the brainstem (spinal V tract), and in the termination of the central processes (spinal V nucleus) weeks before it was detected in the DMNV/SN or ICC of the thoracic spinal cord of the same animals (Table 1; animals: 6099.5, 6100.1–6100.6). Given the relatively early detection of PrP^Sc^ in CN V ganglia reported here and the relative ease of identifying and removing the ganglia, it should be considered as an additional sampling site for the early detection of prions in diagnostic testing. Somatosensation of the oral cavity is also mediated by sensory neurons in CN V ganglia, but they are a different population of cells than those innervating the NC (22); they do not appear to be involved in centripetal transport, as PrP^Sc^ is not detected in CN V ganglia till late in the incubation period following ingestion and is thought to represent centrifugal spread (23). The increasing amount and spread of PrP^Sc^ in CN V-associated structures over time following inoculation (Fig. 2) may contribute to the widespread distribution of PrP^Sc^ throughout the CNS, and it is worth noting that neurons in the spinal V nucleus project to the thalamus, which is affected in most deer and elk infected with a chronic wasting disease (24).

The similarities between the temporal identification of PrP^Sc^ in autonomic CNS structures (ICC and DMNV/SN) following per os (25) and NC infection reported here are consistent with the hypothesis that prions inhaled into the NC are transported into the alimentary tract via the nasopharynx and have the potential to enter the body via the enteric nervous system. The combination of CN V neuroinvasion and the autonomic route (ICC, DMNV, and SN) of neuroinvasion may explain the increased efficiency of the NC route of prion infection compared with the per os route.

## MATERIALS AND METHODS

### Animal care

Procedures involving animals were preapproved by the Creighton University Institutional Animal Care and Use Committee and done in accordance with the *Guide for the Care and Use of Laboratory Animals* (26). Male adult golden Syrian hamsters from Harlan Sprague-Dawley (Indianapolis, IN) were group housed in standard cages with *ad libitum* access to food and water. The results of this study are based on the analysis of additional tissues taken from animals that supplied tissues that were used in a previous publication (2).

### Animal inoculations

Extranasal (e.n.) inoculations were performed as described previously (2). Importantly, the inoculum was placed inferior to the nostrils to avoid the complication of direct inoculation of blood. Hamsters are obligate nose breathers, and the inoculum was immediately inhaled into the NC with no possibility of trauma to the nasal mucosa from the pipette tip. For all inoculations 10 μL of a 1% (wt/vol) bh from either an HY TME-infected hamster (containing 10 ^9.3^ i.c. LD _50_ /g) or an uninfected hamster was used. Hamsters receiving e.n. inoculations were anesthetized with ketamine (120 mg/kg) and xylazine (10 mg/kg) intraperitoneally and 5 μL of inoculum was placed just inferior to each nostril. The inoculum was observed to be drawn into the nasal cavity following ejection from the pipette. The hamsters were then placed supine until anesthesia wore off and they began to move; this took 5–10 minutes.

### Immunohistochemistry

Prion-infected and mock-infected hamsters were anesthetized with isoflurane and perfused transcardially with 50 mL of 0.01 M Dulbecco’s phosphate buffered saline, followed by 75 mL of McLean’s paraformaldehyde-lysine-periodate (PLP) fixative in preparation for IHC to identify the prion protein. The following tissues were immediately dissected and placed into PLP for 24 h at room temperature: V ganglia, SCG, spinal cord, brainstem, and brain. The tissues were embedded in paraffin, cut at 7 µm on a rotary microtome, and mounted onto glass slides. The brain and brainstem were cut in the sagittal plane; both right and left sides were processed identically. Infected and mock-infected tissues for each time point were processed at the same time, using the same reagents. Immunohistochemistry was carried out on the tissue sections as reported previously (2). All tissue sections were deparaffinized and exposed to antigen retrieval in formic acid (minimum 95%) for 10 min at room temperature. The following steps were conducted at room temperature; all incubations were separated by 2–3 rinses with 0.05% Tween in tris-buffered saline. Endogenous peroxidase and non-specific antibody binding were blocked by incubating tissue sections in 0.3% H_2_O_2_/methanol for 20 min, followed by incubation in 10% normal horse serum for 30 min. Visualization of the prion protein was conducted using the avidin-biotin method. Incubation in mouse anti-prion protein monoclonal antibody (Clone 3F4; 1:600, Chemicon, Temecula, CA) was conducted for 2 h, followed by incubation in biotinylated horse anti-mouse antibody (1:600, Vector Laboratories, Burlingame, CA) for 30 min. The sections were placed in ABC solution (1:200, Vector Laboratories, Burlingame, CA) for 20 min and then reacted in a solution containing filtered 0.05% diaminobenzidine tetrachloride (Sigma, St. Louis, MO) with 0.0015% H_2_O_2_. The sections were counterstained with hematoxylin, dehydrated through graded alcohols, and coverslipped with Cytoseal-XYL (Richard Allan Scientific, Kalamazoo, MI). Control sections were processed identically with either the primary or secondary antibody omitted, or with a mouse immunoglobulin G isotype control (1:500; Abcam, Cambridge, MA) in place of the primary antibody.

### Analysis of PrP^Sc^ deposition

To determine the temporal and spatial spread of PrP^Sc^ to the nervous system, structures following exposure to the NC tissue sections were examined using an Olympus BX-40 light microscope and photographed using analysis software (Soft Imaging System, Lakewood, CO). The analysis of tissue sections was performed with the investigator blind to the status of the animals (infected versus mock-infected). For each tissue, the initial detection post-inoculation of PrP^Sc^ deposition was established, as well as the time when all inoculated hamsters demonstrated PrP^Sc^. Tissue sections containing the CN V or SCG from animals at each time point were examined. There were three infected animals at each time point; some structures from some animals were not available (see Table 1). The average number of CN V ganglion sections that was analyzed was 86 (range: 54–100). The average number of SCG sections that was analyzed was 47 (range: 32–72). Two to 5 adjacent brain, brainstem, or spinal cord tissue sections not further apart than 189 µm from animals at each time point were examined. The average number of spinal cord sections that was analyzed was 112 (range: 84–120). The average number of brain/brainstem sections (containing olfactory bulbs, spinal trigeminal tract, spinal trigeminal nucleus, and dorsal motor nucleus of the vagus/solitary nucleus) that was analyzed was 54 (range: 32–78). Hematoxylin and eosin staining on adjacent tissue sections was used to assist in the identification of brain nuclei boundaries (27), and the motor neurons of the DMNV were distinguished from the sensory neurons of the SN based on their larger size and morphology (28, 29).
